# Early modulation of intra-cortical inhibition during the observation of action mistakes

**DOI:** 10.1038/s41598-018-20245-z

**Published:** 2018-01-29

**Authors:** Pasquale Cardellicchio, Pauline M. Hilt, Etienne Olivier, Luciano Fadiga, Alessandro D’Ausilio

**Affiliations:** 1 IIT@UniFe Center for Translational Neurophysiology, Istituto Italiano di Tecnologia, Via Fossato di Mortara, 17-19 Ferrara, Italy; 2 Institute of Neuroscience, Université catholique de Louvain, B-1200 Brussels, Belgium; 3 Section of Human Physiology, Università di Ferrara, Via Fossato di Mortara, 17-19 Ferrara, Italy

## Abstract

Errors while performing an action are fundamental for learning. During interaction others’ errors must be monitored and taken into account to allow joint action coordination and imitation learning. This monitoring relies on an action observation network (AON) mainly based on parietofrontal recurrent circuits. Although different studies suggest that inappropriate actions may rapidly be inhibited during execution, little is known about the modulation of the AON when an action misstep is shown. Here we used single and paired pulse transcranial magnetic stimulation to assess corticospinal excitability, intracortical facilitation and intracortical inhibition at different time intervals (120, 180, 240 ms) after the visual presentation of a motor execution error. Results show a specific and early (120 ms) decrease of intracortical inhibition likely because of a significant mismatch between the observed erroneous action and observer’s expectations. Indeed, as proposed by the top-down predictive framework, the motor system may be involved in the generation of these error signals and our data show that this mechanism could rely on the early decrease of intracortical inhibition within the corticomotor system.

## Introduction

In everyday life, while interacting with others, we continuously infer their intentions^1^ through a combination of bottom-up and top-down processing particularly sensitive to action goals^2–4^. Thus, fast and effective detection of action errors is fundamental for flexible adaptation to other’s behavior and provides essential support for social learning^3^. The literature on action error observation has indicated that different brain regions may be active during error observation. In particular, different parts of the medial prefrontal cortex are active during the observation of unusual actions^5^ depending on whether the observed behavior is intentional or not^6^. At the same time, also simple action error observation elicit an electroencephalographic early error-related negativity (ERN)^7^, similarly localized in medial-frontal structures^8^. However, other studies observed an increase of the P300 component probably associated with a more general monitoring process^9^. The lateral premotor cortex is also activated within both hemispheres, although with a lateralization to the right, during the observation of both correct and erroneous actions^10^. These activations could reflect a matching process between observed actions onto corresponding stored motor representations^11^. In this regard, some studies proposed that social action error detection may rely on our capability in sensing subtle kinematic violations in the observed action^12–14^. According to this view, others’ actions cues are compared to stored internal models of the same action to detect significant deviations^15^. Two different accounts propose two different alternatives to explain how this comparison takes place in the AON (Action Observation Network). The classic AON account suggests a direct matching between observer and actor^11,16,17^ and thus observation of an error should activate the same inhibitory mechanisms at play during error execution^18^. The predictive coding hypotheses suggests that the motor system computes the difference between expected and observed action-related information^19–21^, and thus errors should activate the AON to a greater extent. However, while some studies have shown stronger facilitation in the AON when observing erroneous^22,23^, impossible or uncommon actions^23–28^, other works show greater activity in the AON during observation of correct actions^8,29–31^.

In this study, we investigated the neurophysiological underpinnings of action error processing by focusing on its temporal dynamics. In fact, error processing may involve a cascade of neural events characterized by a temporally fine-grained balance between excitation and inhibition of specific motor programs. To this purpose, we used Transcranial Magnetic Stimulation (TMS) to measure primary motor cortex (M1) cortical and corticospinal excitability^32^, at three time points (120, 180, 240 ms after action error). TMS timing was derived from a previous EEG investigations that shown an EEG error-related negativity (ERN)^8,33–35^ at about 120 ms latency and a correlated ERN feedback component^36,37^ at about 250 ms latency after error occurrence. It is worth noting that 120 ms is also the earliest latency at which corticospinal excitability is modulated by graspable object observation^38^. Specifically, we adopted single pulse (spTMS), short intracortical inhibition (sICI), and intracortical facilitation (ICF) protocols during the observation of picture sequences depicting either correct or erroneous actions. MEPs (Motor evoked potentials) evoked by spTMS provide an instantaneous read-out of the state of the motor system and had been widely used to investigate modulations related to action observation^32,39,40^. Instead, sICI and ICF have rarely been used to investigate AON activity^41–43^, in particular during erroneous actions observation. They differ from the spTMS because they reflect the behavior of distinct populations of inhibitory and excitatory cortical interneurons without affecting spinal circuits^44^. ICF and sICI may reflect the balance between excitation and inhibition mainly mediated by glutamatergic facilitation through N-methyl-D-aspartate (NMDA) receptors^45–47^ and GABA-ergic inhibition through GABA receptors^48–51^.

**Figure 1:**
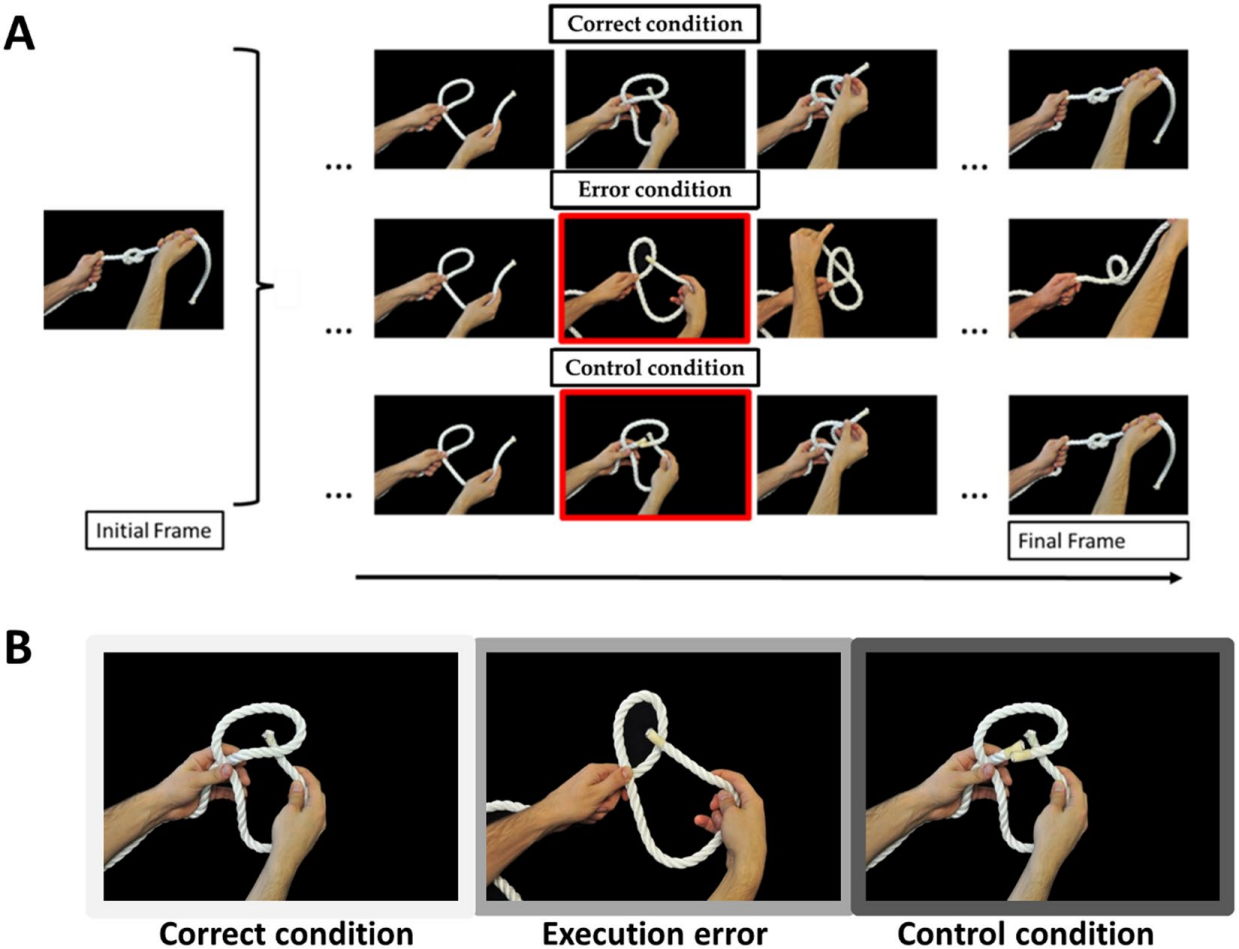
Stimuli and conditions. In panel A, each row represents the timeline of the experimental conditions. For all conditions, the left part of the figure depicts the first frame shown (i.e. the expected final knot). The red squares highlight the frame associated to the error, in both Execution Error and Control conditions. In panel B, each picture shows, from left to right, the Correct, Execution Error and Control conditions.

Action stimuli consisted in knotting actions. While observing someone tying a knot, procedural errors are often conveyed by small visual cues, i.e. the rope passing top-down instead of bottom-up, which however are very important as far as goal achievement is concerned. Interestingly, the use of knots tying, instead of others goal-directed action, reduces the possibility that subjects resort to inner verbalization to rehearse the sequence^52–55^. Knots are indeed very hard to describe verbally and the didactics of knots is almost never based on textual (books) or spoken (online tutorials) material, but rather on visual demonstrations. We used two different type of errors, procedural errors (wrong passage of the rope) and control errors (in which the rope suddenly appears cut in two segments, see Fig. 1A,B).

Considering the direction of the TMS-evoked modulations, two alternative predictions are possible from: (1) the AON account^12–14^ or (2) the predictive coding account^56–59^. The first one suggests an important anatomo-functional overlap between action execution and observation. Following this analogy, increase of inhibition/reduction of facilitation are usually observed in both, volitional inhibition^60–62^ and action error execution^63,64^. Based on the assumption that a strong overlap exists between these two mechanisms^8^, we should see the same pattern of results during the observation of an action misstep (increased inhibition and decreased facilitation). The second one suggests instead that action observation involves the minimization of the sensory prediction error (i.e., Bayesian-like inferences are generated and dynamically compared to the incoming sensory information). These prediction errors propagate through recurrent interactions among the different levels of the cortical hierarchy involved in action perception. The predictive coding framework would then predict greater facilitation and less inhibition in the presence of larger prediction error, as it is the case for the observation an action misstep (decreased inhibition and increased facilitation). The relative balance between local cortical inhibition and facilitation can in principle disentangle which one of the two views is the most effective in explaining how action missteps are incorporated in the representation of other’s action.

## Materials and Methods

### Participants

Nineteen naïve volunteers (8 females; mean age 24 years, range 20–29) participated in the study. All subjects were right-handed, as assessed by the Edinburgh Handedness Inventory^65^. None of the participants reported neurological, psychiatric or other contraindications to TMS^66^. They had normal or corrected-to-normal visual acuity in both eyes and were unaware of the purposes of the study. All of them gave informed consent before the experiment, which was approved by the Ethics Committee of the Ferrara University and conducted in accordance with the ethical standards of the 1964 Declaration of Helsinki.

### Stimuli

The visual stimuli consisted of sequences of eight pictures showing the different steps of an actor (1 male and 1 female) tying a knot (Fig. 1B). All pictures had a uniform black background. Two different actors (1 male, 1 female) recorded from a first-person perspective, were performing two different types of knots. The actors either completed the knot (Correct condition) or did a mistake in executing it (Execution Error condition) by introducing the extremity of the rope inside the loop from top-down instead of bottom-up. This mistake results in the dissolution of the knot and was shown in the fifth picture of the sequence (see Fig. 1B). In the Control condition, we modified the same fifth picture frame by showing the rope cut in two segments (Fig. 1B). This causes the impossibility to achieve the goal as well, but for intrinsic object properties and not for action-dependent factors. In all conditions (Correct, Execution Error, Control condition) the first four frames of each sequence were the same (corresponding to the loop forming, see Fig. 1). Thus, the 3 conditions are perfectly identical until the 5^th^ frame. This choice avoids any prediction from the subjects.

### TMS and electromyographic recordings

Motor Evoked Potentials (MEP) were recorded with a wireless EMG system (Aurion, ZeroWire EMG) from the right *First Dorsal Interosseus* (FDI) muscle by using standard tendon-belly montage with Ag/AgCl electrodes. EMG traces were band-pass filtered (50–1000 Hz), digitized (2 kHz), acquired by a CED power1401 board and visualized with Signal 3.09 software (Cambridge Electronic Design, Cambridge, UK).

A 70 mm figure-of-eight coil connected to a Magstim BiStim stimulator (Magstim Co., Whitland, Dyfed, U.K.) was placed over the left primary motor cortex with the handle pointing backwards at 45° from the midline. As optimum scalp position marked on the scalp of the subjects by using a make-up pencil, was considered the location on the scalp where maximum amplitude MEPs in the FDI were evoked at the lowest possible intensity (hot spot). The resting Motor Threshold (rMT) was assessed by using standard protocols (5 out of 10 MEPs exceeding 50 μV peak-to-peak amplitude^67^), with an inter-stimulus interval of ≅8 seconds.

Three different stimulation protocols were used: Single pulse (spTMS), short interval Intracortical Inhibition (sICI) and Intracortical facilitation (ICF). During the spTMS protocol, a TMS pulse was delivered at the intensity of 120% of the rMT. During the paired-pulse TMS paradigm (ppTMS), sICI and ICF were assessed in accordance with an established protocol^44,68^. The intensity of the conditioning stimulus (CS) was set at 80% of the rMT. Before each experimental session we confirmed that this intensity never induced MEPs in 10 out of 10 repetitions. The test stimulus (TS) intensity was the same as that used in the spTMS session. In the ppTMS the inter-stimuli intervals (ISIs) of 3 ms and 12 ms were used to respectively assess sICI and ICF^44,68,69^. All recorded data are available in Dryad Digital Repository.

### Procedure and experimental design

Subjects were seated on a comfortable armchair. A 17″ LCD computer monitor (1024 × 768 pixels; refresh rate, 60 Hz) was placed at a distance of 58 cm from their frontal plane. Their right hand was placed on a cushion in a relaxed prone position. Before the experimental sessions, participants were familiarized with the visual stimuli. Each trial started with the presentation of a green central fixation cross displayed on a frame depicting the completed knot. After 2000 ms, the knot disappeared and a sequence of pictures was shown. Each picture presentation lasted 200 ms followed by a delay of 800 ms TMS was administered after the fifth picture onset at 3 different delays: 120, 180 and 240 ms.

Participants were instructed to look attentively at each picture sequence and to press a button when they detect something going wrong: wrong knot execution (execution error) or broken rope (control condition). In one third of trials (correct condition), participants did not have to produce any response. Responses were provided with the left hand, ipsilateral to the stimulated motor area, and were recorded by a custom-made response box. Reaction times (RTs) were collected relative to picture onset. In total, 270 trials were randomly presented to every subject: 3 experimental conditions (Correct, Control, Execution Error) X 3 stimulation protocols (spTMS, sICI, ICF) X 3 timings of stimulation (120 ms, 180 ms, 240 ms) X 10 repetitions. Twelve baseline trials for each stimulation protocol (spTMS, sICI and ICF) were recorded at rest (eyes closed, subjects imagining a relaxing landscape^70,71^) at the beginning of the session, and at the end. The presentation of the stimuli, the timing of the TMS pulses and response collection were controlled by Psychtoolbox Version 3.0 (PTB-3), implemented in MATLAB (The MathWorks Inc., Natick, MA, USA).

### Analysis

#### Behavioral data

Incorrect answers or RTs lower than 100 ms or higher than 1000 ms were discarded from the analysis (less than 7% of trials). RTs were analyzed by paired-samples two-tailed t-tests (significance threshold, P < 0.05). The same analysis was applied to responses accuracy.

#### Neurophysiological data

*Preprocessing*: Neurophysiological data were processed off-line by custom-made Signal script (Signal 3.09 software Cambridge Electronic Design, Cambridge, UK). As MEP amplitude we considered the peak-to-peak value (mV). MEPs associated with incorrect answers or with EMG activity in the 50 ms period prior to TMS were discarded from the analysis (less than 10% of total trials number). During spTMS and ppTMS, trials with MEPs lower than 0.05 mV were not considered as proper MEPs and were discarded (less than 2% of total trials number). The average number of trials in each condition was 9.5 trials ± 0.2.

*Baseline modulation*: In the first analysis our aim was to exclude modifications of intracortical and corticospinal excitability during the recording session. We compared baseline spTMS MEPs at the start and at the end of the experiment, with a two-tailed paired t-test. We also verified if sICI and ICF effects were in the direction of inhibition and facilitation, respectively. We ran a repeated-measures ANOVA on MEPs amplitude ratios between ppTMS protocols and the spTMS protocol (mean CS relative to mean TS)^29,68^, using the two protocols (sICI and ICF) and the two baseline as factors.

*Generic action observation modulation*: Furthermore, we verified wether the three TMS protocols were generically modulated by action observation^39^. We compared baseline spTMS MEPs with pooled action observation conditions, with a two-tailed paired t-test. We ran a repeated-measures ANOVA on MEPs amplitude in the ppTMS protocols, using the two protocols (3 ms and 12 ms) and pooled action observation vs. baseline data as factors. As an additional check, we also verified that intracortical inhibition and facilitation was modulated by generic action observation^42,72^. The ratio between ppTMS and spTMS was analyzed with a repeated-measures ANOVA using the two protocols (3 ms and 12 ms) and pooled action observation vs. baseline data as factors.

*Error*-*related modulation*: Finally, we evaluated the effect of the different action observation condition on intracortical and corticospinal excitability modulations. We used a within-subjects repeated-measures ANOVAs, separately for the spTMS and ppTMS protocols. In the spTMS protocol, the dependent variable was MEPs amplitude normalized by the average baseline. The repeated-measures ANOVA included the factors Condition (Correct, Control, Execution Error) and Timing (120 ms, 180 ms, 240 ms). To quantify sICI and ICF action related effects, we expressed MEPs amplitude in the ppTMS sessions in function of the spTMS MEPs amplitude^44,68,73^. For each experimental condition, we then computed a repeated-measures ANOVA using as index of **intracortical** modulation (iMEP) the mean ratio (ppTMS_condition_/spTMS_condition_) over the same mean ratio at baseline (ppTMS_baseline_/spTMS_baseline_), separately for each ppTMS protocols (sICI, ICF). The relationship between the effect found in each condition was then transformed into percentages in multiplying by 100:

1 $$[\frac{(P{P}_{condition}/S{P}_{condition})}{(P{P}_{baseline}/S{P}_{baseline})}]\ast 100$$

A repeated-measures ANOVA was performed on these data with the within-subject factors TMS-protocol (sICI, ICF), Condition (Correct, Control, Execution Error) and Timing (120 ms, 180 ms, 240 ms). All analyses were run by using STATISTICA 9 (StatSoft, Inc.) using Newman-Keuls as post-hoc comparison (P < 0.05) and partial eta-squared for effect size.

## Results

### Behavioral data

Analysis on RTs did not show any significant difference between Execution Error (562 ± 70 ms, mean ± SD) and Control (551 ± 62 ms) conditions (t(18) = 0.64, p = 0.52). Similarly, the accuracy of the responses did not show any significant effect (t(18) = 2.01, p = 0.06) (Execution Error: 78 ± 16; Control: 86 ± 8).

### Neurophysiological data

#### Baseline modulation

Baseline spTMS MEPs recorded at the beginning (mean raw MEP amplitude: 1.66 ± 1.2 mV), and at the end of the experiment (1.58 ± 1.3 mV) were not significantly different (t(18) = 0.48, p = 0.63), confirming no change of corticospinal excitability during the experiment^74^.

The 2 × 2 ANOVA between the TMS protocols (SICI, ICF) recorded in the two baseline (pre, post) revealed a main effect of protocols (F(1,18) = 212,62, p < 0.001; η²p = 0.9), with baseline sICI (mean CS/TS: 0.40 ± 0.36) significantly lower than ICF (1.30 ± 0.54). This result confirms that the two ppTMS protocols elicited the expected intracortical inhibition and facilitation in the baseline recordings. No other main effect (F(1,18) = 0,29, p = 0.59) or significant interaction (F(1,18) = 2,56, p = 0.12) was found confirming no change of cortical modulation during the experimental sessions.

### Generic action observation modulation

During generic action observation (all conditions together), spTMS MEPs amplitude (2.43 ± 1.44 mV) significantly increased compare to baseline (1.61 ± 1.21 mV; t(18) = 3.95, p < 0.001). This result suggests that generic action observation elicits a generic increase of corticospinal excitability, in agreement with previous reports^39^.

The ANOVA on MEPs amplitude during action observation and baseline in the two different protocols (ppTMS 3 ms: action observation: 2.98 ± 1.32 mV, baseline: 2.04 ± 1.13 mV; ppTMS 12 ms: action observation: 0.69 ± 0.73 mV, baseline: 1.11 ± 0.92 mV) showed a significant main effect of TMS-protocol (F(1,18) = 86.51, p < 0.01; η²p = 0.82), with MEPs significantly smaller during the ppTMS 3 ms (mean MEP amplitude: 0.9 ± 0.8 mV) compared to ppTMS 12 ms (mean MEP amplitude: 2.5 ± 1.3 mV). A significant main effect of action observation was also observed (F(1,18) = 25.13, p < 0.01; η²p = 0.58), with MEPs significantly smaller during the baseline (mean MEP amplitude: 1.37 ± 1.1 mV) compared to action observation (mean MEP amplitude: 2.04 ± 1.4 mV). The ANOVA revealed also a significant interaction between TMS-protocol and action observation (F(1,18) = 6.76, p = 0.01; η²p = 0.2). Post hoc analyses evidenced higher MEPs amplitude in the ppTMS 12 ms protocol during action observation compared to other conditions (p < 0.01). In addition, amplitude of MEPs collected during the ppTMS 12 ms baseline was higher than in the ppTMS 3 ms protocols in both conditions (p < 0.01). Similarly to ppTMS 12 ms, MEPs amplitude for the two conditions were significantly different from each other in ppTMS 3 ms (p < 0.01).

The ANOVA on intracortical excitability modulations (ratio between ppTMS and spTMS) during generic action observation and baseline showed only a significant main effect of the protocol (F(1,18) = 153.87, p < 0.01; η²p = 0.8) with higher values in ICF (1.41 ± 0.4) than sICI (0.45 ± 0.2). The action observation main effect was not significant (F(1,18) = 0.618, p = 0.44) nor the interaction (F(1,18) = 3.39, p = 0.08). Although the interaction effect is not significant a trend was reported and is qualitatively visible in the ppTMS/spTMS ratios (ICF: action observation: 1.33 ± 0.29, baseline :1.49 ± 0.54; sICI: action observation: 0.48 ± 0.28, baseline: 0.42 ± 0.28).

#### Error-related modulation

The 3 × 3 ANOVA on spTMS between the condition and TMS timing revealed no significant interaction or main effects (all F < 1.20, p > 0.31) showing no specific modulation of corticospinal excitability induced by error observation.

**Figure 2:**
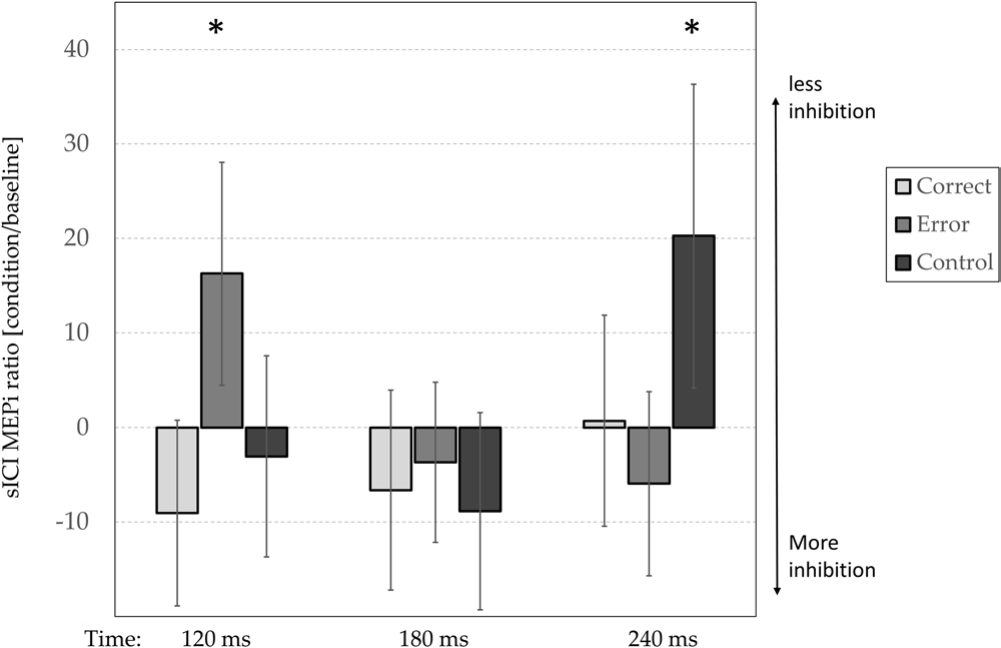
Intracortical inhibition results. Modulation of the iMEP index (ratio of sICI in baseline and conditions, in function of the timing of the ppTMS (120, 180 and 240) in the three experimental conditions (see legend). Vertical whiskers, SEM. Asterisks indicate the significant comparison (Newman-Keuls, P < 0.05). To facilitate the understanding, data presented in this figure are normalized with respect to the mean.

The 2 × 3 × 3 repeated-measures ANOVA on the intracortical modulation index showed a significant main effect of TMS-protocol (F(1,18) = 9,1051, p < 0.01; η²p = 0.3), with iMEPs significantly smaller during the ICF (mean iMEP amplitude: 97% ± 31) compared to sICI (mean iMEP amplitude: 125% ± 48). Moreover, a significant 3-way interaction between TMS-protocol (sICI and ICF), Condition (Normal, Control, Error) and Timing (120, 180, 240) was observed (F(4, 72) = 4,8966, p < 0.01; η²p = 0.2). Post hoc analyses revealed a modulation of iMEPs in the sICI protocol only (Fig. 2). Specifically, iMEPs recorded during the Execution Error were higher at 120 ms (142% ± 51) than in the other timings (180 ms: 122% ± 31, p = 0.010; 240 ms: 119% ± 42, p = 0.009). Moreover, at 120 ms iMEPs recorded during the Execution Error had higher amplitude than the Control and Correct conditions (Correct: 116% ± 42, p = 0.006; Control: 122% ± 46 SD, p = 0.012). A similar effect was found for the Control condition but at different Timing. The iMEPs values for the Control condition are higher at 240 ms (146% ± 69) compared to other Timing (120 ms: 122% ± 46, p = 0.003; 180 ms: 116% ± 45, p < 0.001). At this timing (240 ms), Control iMEPs had higher amplitude than the Correct and Execution Error conditions (Correct: 126% ± 48, p = 0.010; Execution Error: 119% ± 42, p = 0.001). No other main effect or interaction was significant. Summing up, these results point out a significant reduction of intracortical inhibition at 120 ms for the Execution Error and at 240 ms for the Control conditions (Fig. 2).

## Discussions

Action understanding is the building block of many important social cognitive skills, such as communication, imitation, intention understanding, learning and empathy^75^. The relevance of predicting the consequence of other’s actions to understand “what” is happening has been extensively discussed at a theoretical level^76^. However, less is known about the neural mechanisms used to cope with the rather frequent circumstances where these predictions are wrong because an error happens in the observed action.

In this study, we aimed at investigating whether and how the motor system is sensitive to the observation of action missteps. We demonstrated an early (120 ms) reduction of inhibition for the observation of a motor execution error, while the control error elicited a similar effect but with a longer latency (240 ms). A similar biphasic modulation has also been shown for corticospinal excitability during action observation^77^. In Barchiesi and Cattaneo (2013), the early corticospinal modulation followed the automatic mapping between action execution and observation properties, whereas later effects were driven by the recent history of visuomotor associative learning. In general, our results support the hypothesis that early and late motor activations induced by action observation may reflect two distinct mechanisms. Our early effect is associated to the presentation of a motor execution error. A delay of 120 ms was shown to be enough to activate the motor system during graspable object presentation^38^. This condition requires that the observer maps the functional relationships between hands and rope positions to derive the presence of an error. The late effect instead, is triggered by a cut in the rope which, independently from the action performed by the actor, do not allow the successful conclusion of the action. The detection of this latter deviation from the expected action outcome, may require access to strategic and abstract reasoning regarding the feasibility of the action plan, that only later translates into the intracortical modulation of the motor cortex^78^.

Interestingly, using single and paired-pulse TMS protocols, we could investigate changes in corticospinal excitability as well as intracortical facilitatory (ICF) and inhibitory (sICI) circuits while participants were being presented with different types of errors. Notably, these indexes have already proven to be more sensitive than the MEPs recording during spTMS in detecting weaker sensorimotor associations^79^. Corticospinal excitability reflects the effect of inhibitory and excitatory inputs to the descending corticospinal pathway. The sICI and ICF reflect distinct neurophysiological mechanisms^45,80^. sICI is associated to the activation of low threshold inhibitory interneurons in M1 mediated by gamma-aminobutyric acid (GABAa) receptors^48,49,51^. The ICF more likely reflects the work of glutamatergic excitatory M1 circuits involving N-methyl-D-aspartate (NMDA) receptors^45^. ICF, but not sICI, is thought to be influenced by the activation of long-range connections originating from remote brain regions^45,81^. Hence, our results reveal an early modulation of GABA-ergic inhibition in the motor system, driven by action error observation. Effects were observed for sICI but not for ICF, suggesting that the neural mechanisms involved in detecting action execution errors mainly consist in the modulation of intracortical inhibitory circuits. The lack of ICF effects is in line with previous studies showing no agreement on ICF modulations during action observation^42,72,82,83^. Similarly, previous works show that volitional inhibition in action execution does not affect ICF measures, but only sICI^84^.

Moving to the functional meaning of our results, according to the standard AON account, observing an action causes the reactivation of the same motor circuits in the observer’s brain^17^. However, our results seem to go in an opposite direction. In fact, peri-movement modulation of sICI is associated to the mechanism by which voluntary movement is gated on and off. Indeed, the magnitude of sICI is reduced just before voluntary contraction^64^, increased before its cessation^85^ and is somatotopically specific^86^. TMS studies of action observation have shown an increase of excitation in terms of corticospinal excitability^39^ paralleled by a decrease in sICI^42,72^. These findings parallel the local intracortical excitatory and inhibitory dynamics observed during actual action execution by shifting the balance towards greater local excitation^42,72^. As a consequence, observing action errors would set in motion the neural cascade of events that normally occur during the suppression of erroneous voluntary movements. For instance, in the stop-signal task a decrease in corticospinal excitability and an increase of sICI^60–62^ is commonly observed. The magnitude of sICI acting on the agonist muscle increases also in the No-Go phase of a Go/No Go reaction time task^84^, and in a countermanded reaction time task when the prepared movement is successfully retained^62^. This sICI increase was also present in others muscles, not engaged in the action^87^ and may prevent unwanted activations^80,84^.

Our results, however, show that when an action error is detected, a decrease in inhibition rather than an increase is present. This is the opposite of what we would expect from a complete functional match between action execution and action observation processes. The predictive coding account^56,57^, which has also been extended to explain mirror-like activities^88,89^, could offer some insight. This model suggests that the brain uses all available information to continuously predict forthcoming events and reduce sensory uncertainty by dynamically formulating perceptual hypotheses^90^. The formulation of perceptual hypotheses and their verification against incoming data, is fundamentally constrained by knowledge about the neural and biomechanical organization of movements^91,92^. This process occurs at all levels of the cortical processing hierarchy and is hypothetically instantiated in two types of computational units^76,93^, representation and error units. While the representation units encode the predictions based on prior information, the error units compare the incoming signals with the predictions conveyed via the representation units. The discrepancies between predictions and input signals generate a prediction error signal. This prediction error signal updates the generative model at the next level of the cortical hierarchy and is consequently a critical component of the predictive mechanism^76,94^.

In this context, the main function carried out by the AON could be that of computing prediction errors based on visually perceived actions and to propagate them throughout the motor hierarchy^28,95–100^. Therefore, greater AON activities should correspond to either greater prediction errors or errors whose implications extend across the motor hierarchy.

Remarkably, our study significantly expands on these aspects by showing that observing erroneous actions does not elicit increased inhibition as it would be predicted by the classic view about motor mirroring of other’s action. Instead, the release from inhibition could be explained by the greater mismatch with respect to the generated top-down predictions. Action errors, as the one we investigated here, provide relatively small visual cues to disentangles errors from correct events. Nevertheless, these visual cues contain significant informative messages since the implications of such small and local differences directly propagate throughout the action hierarchy making it readily clear that the action goal will not be achieved.
